# Annotations

**Published:** 1907-12-21

**Authors:** 


### ANNOTATIONS.

### Medical Students and their Critics.

The medical profession in certain of its activities forms a stock subject for censure and criticism by various authorities in the columns of some of our lay contemporaries. We have no complaint to make on the score of publicity, nor have we any doubt of the honesty and good intentions of our censors. Possibly at times they are lacking in full and correct information, and they not infrequently display a tendency to erect large generalisations on a somewhat scanty basis of fact. Yet we by no means desire their suppression, even if such an occurrence were possible, and we recognise the advantage of being criticised from the outside although such criticism often contains a measure of injustice. Recently there have been published some distinctly scathing censures on the noisy and disorderly protests, organised by some medical students, against an inscription on a statue erected at Battersea to the memory of a dog which was killed, it is alleged, in the process of some vivisection experiments. Certainly the profession generally will neither defend nor sympathise with any infringement of public order, and it may be allowed that the proceedings of the students have been neither wise nor dignified. But to found upon these proceedings a charge against the character and disposition of medical students generally, or to inquire whether such occurrences do not cause anxiety in regard to the fitness of medical men to take charge of "the children and the poor of our land," comes perilously near to bathos. In promoting disorder the lads were certainly at fault, but something more than a students' row or two is necessary to sustain the impeachment of a profession. And even a Puritanical critic must allow that provocation existed, and that the students were stirred, although possibly unnecessarily, by loyalty to their teachers and their *alma mater*.

### Pneumococcus Infections.

The recognition of micro-organisms and their products as the causes of many diseases has revolutionised medical opinion and practice in numerous directions, and the revolution is not yet complete. It will be for those to whom in time will fall the duty of writing the medical history of the past thirty years to estimate the full scope of the change which has thus been effected. An illustration of the change here alluded to may be found in the paper recently submitted to the Medical Society by Professor Wm. Osler. No doubt there have been at times suggestions that pneumonia is an infectious disease, and certain epidemic outbreaks, particularly in the North of England, have more than once attracted attention. But for the most part the disease, until recent years, was regarded as a local affection of the lungs, and without risk to those in attendance on the patient. Now the picture is presented in very different colours. The pulmonary consolidation is defined as but one evidence of a generalised infection which may produce many other inflammatory manifestations, or may in occasional instances exist as a toxæmia or septicæmia without recognisable local disturbance. From this position in the individual patient the question arises of the risk to the community, and Professor Osler does not hesitate to say that preventive medicine in regard to pneumonia has to deal with a "serious situation," and that the problem is one fully as deserving of study as cancer or tuberculosis. To these statements have to be added the fact that pneumonia is a disease which is increasing in frequency, more particularly among town dwellers, and that the pneumococcus exists in large numbers in the sputa of the patients. Thus it becomes a question whether methods of isolation should not be adopted. Certainly it would seem right to disinfect the sputa and, so far as possible, the mouth and upper air-passages. For the latter an alcoholic solution of sodium bicarbonate and sodium chloride with glycerine, and made as strong as the patient can bear it, is advised.

### Society for the Study of Disease in Children.

The special discussion arranged by this Society as an annual feature of its work was held on December 13, and was devoted to the subject of "Congenital Syphilis." Mr. R. Clement Lucas, who read the introductory paper, gave an effective sketch of the modern position of the subject, and suggested a number of points on which an authoritative expression of opinion is to be desired; he also urged that every care should be taken to secure a complete investigation before applying the diagnosis to any individual case. Mr. Geo. Pernet deplored the use of the term "hereditary" syphilis, arguing that the evidence showed that infection was never direct from the sperm to the germ cell, but that the fœtus always received the disease from the mother; he also expressed his conviction of the accuracy of Colles' law. A most lucid and practical contribution to the discussion was made by Dr. H. G. Adamson, who discussed the differential diagnosis of skin eruptions affecting the "napkin region" in infants; his description was effectively illustrated by diagrams taken from actual cases. Mr. Sydney Stephenson described the evidences of congenital syphilis met with in the eye, and discussed these on the basis of statistics prepared from his own cases. He pointed out that interstitial keratitis not very infrequently develops after some local injury or after an illness which depresses the patient's general health, and that occasionally ulceration of the cornea occurs in this affection. He also quoted instances in which interstitial keratitis was the result of acquired syphilis, and contended that the affection is occasionally due to tubercle or other general infection. Dr. Leonard Guthrie dealt with the occurrence of interstitial nephritis in infants, and argued that though this is sometimes syphilitic, there are other cases in which no evidence of such a cause can be obtained. In closing the debate Dr. Geo. Carpenter reviewed the influence of congenital syphilis on the bones and viscera, and showed some interesting specimens of cranio-tabes and Parrott's nodes. The meeting was a very successful one, and the record of the discussion will be recognised as a valuable contribution to an important subject.

